# A Comparison of Two Commercial Swim Bench Ergometers in Determining Maximal Aerobic Power and Correlation to a Paddle Test in a Recreational Surfing Cohort

**DOI:** 10.3390/sports7110234

**Published:** 2019-11-11

**Authors:** James Furness, Linley Bertacchini, Lisa Hicklen, Dane Monaghan, Elisa Canetti, Mike Climstein

**Affiliations:** 1 Water Based Research Unit—Bond Institute of Health and Sport, Bond University, Gold Coast, QLD 4226, Australia; linley.bertacchini@student.bond.edu.au (L.B.); lisa.hicklen@student.bond.edu.au (L.H.); dane.monaghan@student.bond.edu.au (D.M.); ecanetti@bond.edu.au (E.C.); michael.climstein@scu.edu.au (M.C.); 2 School of Health and Human Sciences, Southern Cross University, Bilinga, NSW 2480, Australia; 3 Physical Activity, Lifestyle, Ageing and Wellbeing, Faculty Research Group, Faculty of Health Sciences, The University of Sydney, Lidcombe, NSW 2141, Australia

**Keywords:** aerobic power, swimbench, VASA, surfing, SwimFast, Cardiorespiratory fitness, VO_2peak_

## Abstract

The recent addition of surfing to the Tokyo 2020 Olympic Games has fueled a surge in commercial and research interest in understanding the physiological demands of the sport. However, studies specific to maximal aerobic testing of surfers are scarce. Therefore, the primary aim of this study was to compare two commercially available swim bench (SWB) ergometers in the determination of maximal aerobic capacity in recreational surfers. A secondary aim was to correlate (independent of one another) the two ergometer findings of VO_2peak_ to the time taken to complete a water-based 400-m paddle test. This cross-sectional study consisted of 17 recreational surfers aged between 18–58 years. Participants were randomized to either the SwimFast ergometer or VASA ergometer and tested for maximal aerobic capacity, followed by a 400-m paddle test. There were no significant differences between the two SWB ergometers in the determination of relative VO_2peak_ (mean difference 0.33 mL/kg/min; 95% CI −1.24–1.90; *p* = 0.66). Correlations between VO_2peak_ obtained from maximal paddling effort on the SwimFast and the VASA and the 400-m paddle test (total time (s)) showed a negative significant correlation *r* = −0.819, *p* = 0.024; *r* = −0.818, *p* = 0.024, respectively. Results suggest that either ergometer (SwimFast or VASA) can be used to determine peak aerobic capacity within a recreational surfing cohort. The significant correlation of the two SWB ergometers and the 400-m paddle test suggest that the 400-m paddle test may be a suitable field-based method of determining aerobic capability. Collectively, these preliminary findings provide initial evidence for similarities in VO_2peak_ on two commercial ergometers and their correlations with a field-based test. However, further research is needed with a larger sample size and inclusive of competitive surfers to provide robust findings which can be generalized to the surfing population.

## 1. Introduction

For the first time in history, 20 female and 20 male surfers will compete for Olympic medals in Japan [1]. The recent addition of surfing to the Olympic Games in Tokyo 2020 has fueled a surge in commercial and research interest in understanding the physiological demands of the sport. This has made evident the need for suitable and specific testing protocols for these athletes, coaches, and sport scientists in a laboratory- and field-based environment. Fundamentally, surfing can be broken down into sitting, paddling, and wave riding, with paddling having the greatest activity requirement [2,3,4]. This has been further reinforced with global positioning system (GPS) data which indicated that approximately 50% of a 30-min competitive surfing session involved paddling in varying durations and speeds [3]. Furthermore, within a recreational surfing session, paddling was shown to represent 42.6–44% of the total time [4,5], with the majority of these paddling bouts (60%) ranging from 1 s to 20 s long [5]. This intermittent manner of surfing accentuates the importance of both the aerobic and anaerobic energy systems, suggesting that a high level of aerobic fitness is essential for surfing [6].

The physical demands of surfing require athletes to have high muscular endurance, anaerobic power of the upper torso, excellent cardiorespiratory fitness, and the ability to recover rapidly [7]. Given the previously stated physiological demands needed while paddling, specificity of aerobic fitness testing procedures for surfers is warranted. Farley et al. [8] investigated heart rate (HR) of surfers during competition. The authors reported a mean HR of 139 beats per minute (bpm), equating to approximately 65% of the individuals age-predicted HR maximum, with peak HR reaching up to 190 bpm, which was 87% of the individuals age-predicted HR maximum. The variation in moderate to high HRs demonstrate the contribution of both the aerobic and anaerobic energy systems and highlights the need for high level upper body aerobic fitness [8].

Aerobic and anaerobic capacity are assessed by the determination of maximal oxygen consumption (VO_2max_) and peak oxygen consumption (VO_2peak_). This is achieved through the quantification of O_2_ and CO_2_ concentrations and expired air volumes in expired air collected through an incremental effort to maximal (or volitional exhaustion). Treadmills and cycle ergometers are routinely used to provide the incremental loads for such tests. Multiple studies have been conducted [7,9,10,11,12] to obtain the physiological profiles (VO_2max_, peak heart rate, VO_2peak_, and peak blood lactate) of athletes across a variety of water-based sports. The devices used include cycle ergometers [9], treadmills [6,7,13], tethered swimming [7,9,10], swim flume [14], and swim ergometers [2,6,8,15]. VO_2max_ and VO_2peak_ results obtained utilizing cycle ergometers or treadmills for data collection in a surfing population lack specificity to surfing due to the sport’s dominance of upper body demands. The significant differences in oxygen consumption and utilization between leg and arm work during maximal and submaximal exercise [16] support the use of a site-specific ergometer. Therefore, upper-body-specific devices, such as the swim bench ergometer, swim flume, and tethered swimming, are the preferred methods of providing incremental load for the assessment of aerobic and anaerobic capacity of surfers.

Previous studies have conducted research specific to prone ergometers among the surfing population, including the Dansprint kayak [8], VASA swim bench ergometer [2,12,14], Monark cycle ergometer [7], SwimFast ergometer [6], and modified kayak ergometer [17,18]. Collectively, differences in testing equipment have limited the ability to draw direct comparisons within the existing literature on the physiological profiles of surfers. Even in studies which have utilized swim bench ergometers, variation exists in the type and model of equipment. For example, Furness et al. [2] utilized a wind-braked swim ergometer developed by VASA (Vasa, Inc., Essex Junction, VT, USA), while Khundaqji et al. [6] utilized a swim bench ergometer developed by SwimFast (KayakPro SwimFast, Miami, FL, USA). Although these studies utilized swim bench ergometers, albeit from different manufacturers, there was a difference of approximately 20% in mean VO_2peak_ values.

Despite variations within testing protocols, information regarding a surfer’s aerobic capacity needs to be obtained and reviewed to match the demands of sport. To date, research specific to a surfing population has determined VO_2peak_ scores using laboratory-based equipment [2,6,7,8,12,14,15], thus limiting access to coaches and athletes within the surfing population. Access to these physiological results would greatly benefit the athletes in their aerobic exercise training. One alternative to this problem is the utilization of field-based tests, such as the 400-m paddle test [19] or multistage shuttle swim test [10]. Field-based tests have been developed to assess aerobic fitness while allowing a large group of subjects to be tested with relative ease and minimal cost [10].

Rechichi et al. [10] identified a significant positive correlation (*p* < 0.001, *r* = 0.83) in water-polo players between VO_2max_ data obtained from the multistage swim test and a tethered swim protocol [10]. Such correlation is yet to be established in the surfing population. To the authors’ knowledge, only one field-based study has used the 400-m paddle test within a surfing population [19]. Although this study highlighted the ability of the 400-m paddle test to discriminate between surfing ability (competitive and recreational) within a surfing population, there is still no information on how aerobic results from this field-based test compare to a laboratory-based test. 

Therefore, the aim of this study was to compare two commercially available swim bench ergometers in the determination of maximal aerobic fitness in recreational and competitive surfers. The secondary aim of this study was to correlate (independent of one another) the two ergometer findings of VO_2peak_ to a water-based 400-m paddle test. It was hypothesized that VO_2peak_ data would not differ significantly between the two ergometers. Second, it was hypothesized that VO_2_ findings obtained by each ergometer would correlate with the 400-m paddle test.

## 2. Materials and Methods

### 2.1. Participants

This cross-sectional study included recreational (n = 17) surfers aged 18–58 years. Participants with a minimum of 12 months surfing experience, who were currently surfing and whose main form of main form of physical activity was surfing, and who were not competing at a higher level than local club were considered recreational surfers. Participants were informed of the associated risks and benefits of the study prior to providing informed consent along with a standardized adults pre-exercise screening tool from Exercise and Sport Science Australia. This screening tool was used to identify any participants which may have underlying medical conditions that put them at risk during maximal exercise and therefore would be excluded from participation in the study. Participants were provided an explanatory statement for the testing protocol. Participation was completely voluntary and any participant could withdraw at any time without penalty. Recruitment occurred through poster advertisement at Bond University and Southern Cross University (Gold Coast) as well as investigators’ surfing networks. All testing of participants was conducted at the Bond University Institute of Health and Sport in the Water Based Research Laboratory. Ethics were granted through the Bond University Human Research Ethics Committee (JF00951) prior to commencement.

### 2.2. Anthropometrics

Anthropometric data, including height (cm) (EcoMed Seca, Hamburg, Germany) and body mass (kg) (Wedderburn, WM204, Sydney, Australia), were collected. Height was measured to the nearest 0.1 cm and body mass was measured to the closest 100 g with minimal clothing using a standard medical balance scale.

### 2.3. Participants Surfing Demographics

Participant surfing history was collected and reported through the Surfing Medicine International Surf Years Calculator application on a mobile phone. This app produces a numerical value of an individual’s lifetime surf exposure, referred to as surf years, and incorporates additional surfing hours during holidays and time periods where there is reduced participation in surfing [20]. A single surf year is defined as five hours spent in the water surfing every week for one year.

### 2.4. Aerobic VO_2peak_ Testing

Testing was conducted by three experienced exercise scientists under the supervision of a senior researcher with expertise in maximal aerobic and anaerobic testing of recreational and competitive surfers. All subjects were tested on the VASA swim bench (SWB) ergometer, SwimFast SWB ergometer, and an optional 400-m surfboard paddle test.

The VASA and SwimFast ergometers are two commercially available. swim bench ergometers. Both ergometers utilize an adjustable resistance air-braked flywheel. However, the SwimFast bench allows the upper body to rotate to both sides a maximum of 30°. Additionally, the SwimFast has adjustable arms (length and width), which are designed to accommodate different swim strokes. These key variations may result in physiological differences when using either device for exercise testing or training

To minimize systematic bias, the order of aerobic VO_2peak_ testing on each device (i.e., VASA SWB ergometer or SwimFast SWB ergometer) was randomized by a computer-generated system. All tests were conducted on three separate occasions with a minimum of 48 h of rest between tests.

### 2.5. SwimFast and VASA Ergometers

Prior to commencing the incremental exercise testing, subjects were provided with standardized instructions on the use of the SwimFast SWB ergometer (KayakPro SwimFast, Miami, FL, USA) and VASA SWB ergometer (Vasa, Inc., Essex Junction, VT, USA). Oxygen consumption was analyzed using an automated gas analysis system (Parvo Medics, TrueOne®, 2400, Sandy, UT, USA), which was calibrated with laboratory grade standard gasses (O_2_ and CO_2_) prior to each test.

The SwimFast SWB ergometer familiarization included appropriate fitting of the mouthpiece, headpiece, and ergometer measurements for comfort, which included bodyboard position length, leg support position, and hand paddle fitting. The stability at the chest was unlocked, meaning subjects needed to control their rotation through strokes. The paddle handles were set at 15 degrees (the angle at which the arms are set at) and the resistance at level 1 (this setting controls how much air is drawn into the cage for each stroke).

Expired gases data were collected for 1 min at rest with the participant lying prone on the ergometer with the mouthpiece and headpiece in situ to determine baseline information. Subsequently, subjects completed a 1-min warm-up, which consisted of paddling below 10 Watts (W). Following the warm-up, each participant completed graded maximal exercise testing commencing at 10 W with increments of 10 W increases every minute. 

**Figure 1:**
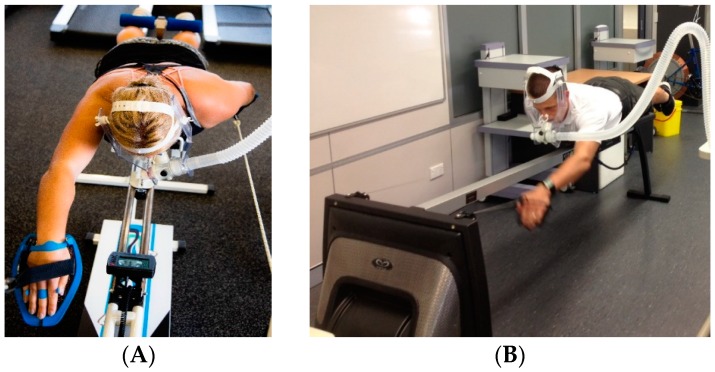
Prone set up position on SwimFast (**A**) and VASA SWB (**B**) with headpiece, mouthpiece and arm paddle.

This incremental test was adapted from a previously validated protocol by Furness et al. [2]. The testing termination criterion was based upon the ACSM guidelines for exercise testing and prescription [21]. Testing was terminated if any of the following criteria were met: Age-predicted maximal heart rate was exceeded, oxygen consumption did not increase concurrently with an increase in power output, required power output was not maintained for greater than 10 s, volitional exhaustion was achieved, or any symptoms of chest pain or discomfort were expressed by the participant. HR was collected every 30 s throughout testing via telemetry (Polar H7 Bluetooth HR Sensor), displayed on an iPad using the Polar Flow application., and recorded manually on a participant data collection sheet. Participants were encouraged to continue to paddle for approximately 1 min at the termination of the test at a low workload as a cool down. An illustration of the experimental set up on both ergometers is provided in Figure 1.

### 2.6. 400-m Paddle Test

**Figure 2:**
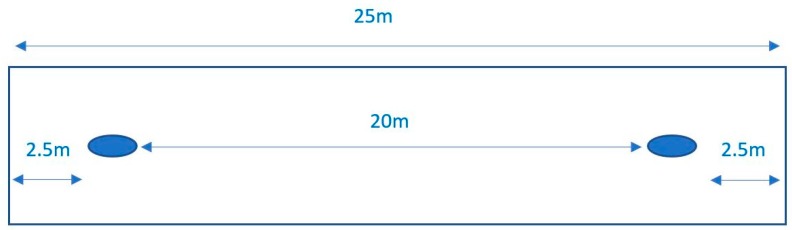
Schematic diagram of pool arrangement for the 400-m paddle test.

**Figure 3:**
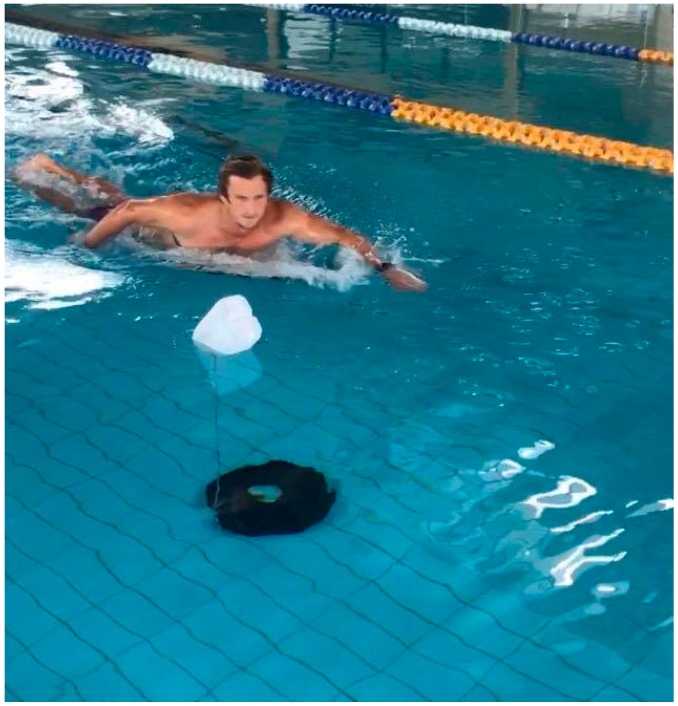
Participant completing 400-m paddle test.

Prior to commencing the 400-m paddle test, participants were provided with standardized instructions on the test. Two buoys were placed 2.5 m from each end of a 25-m lap pool, creating a 20-m distance for test completion of a single lap. A total of 20 laps were completed to reach 400 m (see Figure 2 and Figure 3). Testing was conducted in an indoor pool, with ambient (22.2 °C) and water temperature (29.1 °C) and fluctuations of about 1 °C due to humidity. Participants performed the test on their selected short surfboard where measurements of height, width, thickness (collected from participant’s board), and board volume (calculated via equation: 58% of length (cm) × width (cm) × thickness (cm)) were collected. Prior to commencement of the shuttle test, participants were given a standardized warm-up to complete 4 m × 20 m shuttles at 50%, 60%, 70%, and 80% of the participant’s perceived maximal paddling effort. At completion of the warm-up, the participant was given a 1-min rest period prior to the beginning of the test. To begin the test, participants were assisted in aligning their surfboard with the buoy and were signaled to commence the test.

In order to reduce bias of pacing strategies, participants were encouraged to complete the test as fast as possible. Lap times were recorded and documented at each 20-m lap completion as the tip of the board reached the end buoy inclusive of the turn. Data was collected with each individual lap time obtained as well as the total time taken to complete the 400 m. Average HR and maximum HR were collected via a Polar FT1 Bluetooth HR sensor and watch.

### 2.7. Statistical Analysis

Descriptive statistics, including means (M), standard deviations (SD), and ranges, were calculated for key performance variables (VO_2peak_, HR_peak_, percent of age predicted HR_max_, and peak aerobic power). Normal distribution of the data was confirmed through a Shapiro–Wilk test and visual inspection of box plots, normal Q–Q plots, and frequency histograms. A paired sample t-test was used to determine whether there was a statistical mean difference between participants VO_2peak_ scores on the Swim Fast and VASA SWB ergometer. Alpha was set at *p* < 0.05 *a priori* to determine significance. The level of agreement between the two ergometers was determined using a one-sample t-test and was also presented through a Bland–Altman plot with the associated 95% limit of agreement. Mean difference between measures ±1.96 × SD, was used as the formula [22]. A Bivariate Pearson’s correlation analysis was used to determine correlation between VO_2peak_ scores and the time to complete the 400-m paddle test. All statistical analyses were performed using SPSS (Version 25.0; IBM Corp., Armonk, NY, USA).

## 3. Results

### 3.1. Physical Attributes of Recreational Surfers and Key Performance Variables

Physical attributes and surfing experience for the 17 recreational surfers are depicted in in Table 1. Descriptive statistics, including means (M), standard deviations (SD), and ranges, were also calculated for key performance variables (VO_2peak_, HR_peak_, respiratory exchange ratio (RER), and peak aerobic power) and are presented in Table 2.

### 3.2. Comparative Analysis between the SwimFast and VASA Ergometer

A paired-sample t-test was used to determine whether there was a statistical mean difference between participants aerobic capacity (VO_2peak_), heart rate, and peak power output between the two ergometers. Results of the paired samples t-tests are presented in Table 2 and demonstrated no significant differences for all key performance variables.

### 3.3. Agreement Between Ergometers (SwimFast and VASA)

**Figure 4:**
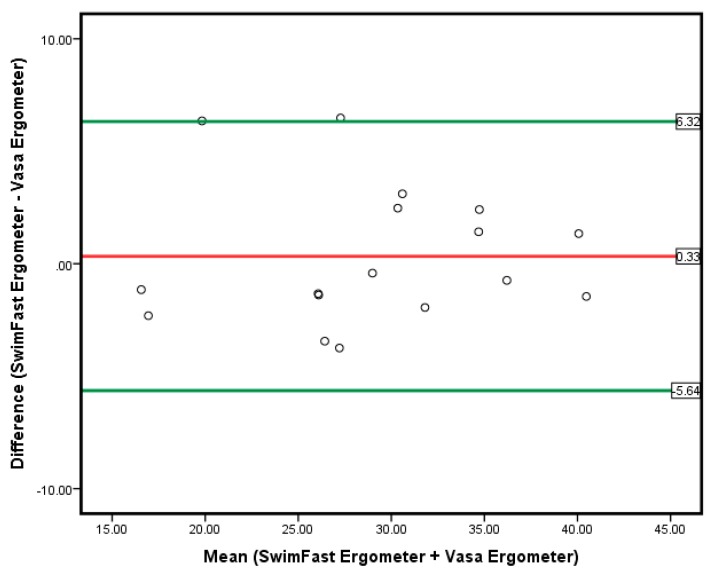
Bland–Altman Plot displaying mean difference between two ergometers with the associated 95% limit of agreement for VO_2peak_.

A graphical representation of the agreement between the SwimFast SWB ergometer and the VASA SWB ergometer VO_2peak_ values was displayed using a Bland–Altman plot and the associated 95% limit of agreement (6.34 to −5.64 mL/kg/min) and overall mean difference (0.33 mL/kg/min) (see Figure 4).

### 3.4. Correlation between Field-Based and Laboratory Tests

A total of nine participants completed both the laboratory and field-based tests. A scatterplot was conducted initially to view datapoints for both the laboratory-based and 400-m paddle tests. Visual inspection of the scatterplot for VO_2peak_ on SWB ergometer and the 400-m paddle test time revealed two datapoints that were clearly outliers. Further inspection of each outlier datapoint revealed that those participants were on a shorter board (board height = 167.64 cm) compared with average board height (185.2 cm). Given the clear differences seen on visual inspection and the differences in board dimensions, removal of the outliers was warranted as described by Laerd Statistics [23].

**Figure 5:**
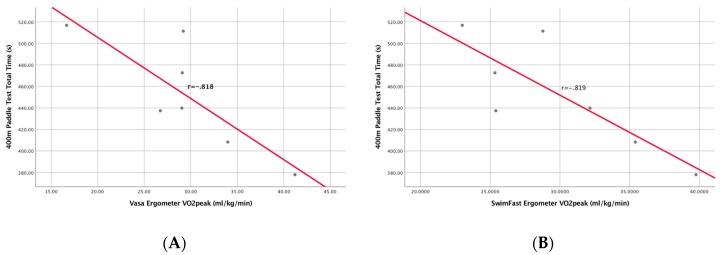
Pearson’s correlation between the 400-m paddle test total time: (**A**) The VASA SWB ergometer; (**B**) the SwimFast SWB ergometer (n = 7).

Pearson’s rank order correlation test between the time taken to complete the 400-m paddle test and the VO_2peak_ determined using the VASA ergometer showed a significantly negative correlation r = −0.818, N = 7, *p* = 0.024 (Figure 5a). VO_2peak_ determined on the SwimFast ergometer and the 400-m paddle test time were also significantly negatively correlated r = −0.819, N = 7, *p* = 0.024 (Figure 5b).

## 4. Discussion

The purpose of this study was to compare two commercially available swim bench ergometers in the determination of maximal aerobic fitness in a recreational surfing cohort. The secondary aim of this study was to correlate (independent of one another) the two ergometer findings to a water-based 400-m paddle test. These aims satisfied our hypothesis that VO_2peak_ data did not differ significantly between the two commercially available ergometers. The study also revealed that VO_2peak_ scores obtained by each ergometer correlated with the 400-m paddle test.

The participant demographics, including anthropometric measurements and surfing experience, are in accordance with current literature specific to surfing cohorts [2,6]. This study identified that there was no significant difference between mean VASA SWB ergometer VO_2peak_ and SwimFast SWB ergometer VO_2peak_ (see Table 2). These findings confirmed the original hypothesis. However, it could be assumed that peak aerobic power would be higher in the SwimFast ergometer than the VASA ergometer given the fact that the SwimFast ergometer allows for 30° rotation of the upper body whilst paddling. The premise for this assumption is due to the torsional rotation allowed by the SwimFast might lead to increased use of the thoracic musculature, resulting in an increase in oxygen consumption, which leads to a higher VO_2peak_. As this was not the case, it can be assumed that the additional rotation of the SwimFast did not contribute to significant increases in oxygen demands. The VO_2peak_ results using the SwimFast and VASA within the current study (28.24 ± 7.28 to 28.91 ± 7.16 mL/kg/min, respectively) are in agreement with previous research conducted on recreational surfers using the VASA and SwimFast SWB ergometer (VO_2peak_ scores ranging from 28.91 ± 7.16 to 37.41 ± 9.08 mL/kg/min) [2,6,14]. Given the current similarities in VO_2peak_ scores across both ergometers, either device may be used to test VO_2peak_ in a recreational surfing cohort.

The current study is the first to compare relative VO_2peak_ of laboratory-based test and a 400-m paddle test [19] in a surfing cohort. Findings showed that there was a large negative significant correlation between the VO_2peak_ score obtained on the SwimFast and VASA SWB ergometer and the 400-m paddle test time. Results supported that as relative VO_2peak_ increased, 400-m paddle test time decreased (see Figure 5). The physiological demand of the maximal aerobic capacity in a laboratory-based test correlated with the endurance demand of the 400-m paddle test. Similar associations have been established between laboratory and field-based tests in other water-based sports. Rechichi et al. [10] identified a significant correlation between a multistage shuttle swim test, tethered swimming test (VO_2max_), and a 400-m swim test in water polo players. It needs to be noted that Furr et al. [14] revealed a significant difference in VO_2peak_ scores between a flume-based study and the VASA swim bench ergometer, revealing an 11% greater difference when using the flume-based testing procedure. This highlights the additional physiological demands of the water and the need to utilize testing procedures other than swim bench ergometers.

The 400-m paddle test time (n = 7; 452 s ± 51.5) in the current study is comparative to the only other published study [19] using the 400-m paddle test. Farley et al. [19] used the 400-m paddle test to determine the test’s discriminative validity. The study included recreational surfers and revealed significantly longer paddling times and slower speeds in the recreational cohort when compared with the competitive surfers (competitive: 350 s and recreational: 450 s).

### 4.1. Clinical Applications

There are two key clinical applications of the findings from the current study. First, the similarities in relative VO_2peak_ scores across both SWB ergometers results emphasize that either of the SWB ergometers can be effectively utilized when testing maximal aerobic capacity in a recreational surfing cohort. It could be suggested that if surfers, coaches, or sport scientists only have access to either SWB ergometer, they will obtain suitable data related to maximal aerobic capacity. Given that the 95% limit of agreement range from 6.34 mL/kg/min to −5.64 mL/kg/min, the authors recommend using the same SWB ergometer for follow-up testing on the same surfer. 

Second, the results showed a negative significant correlation between the relative VO_2peak_ scores for both ergometers and the 400-m paddle test time. The current findings are noteworthy due to the limited accessibility and cost of using a laboratory environment. A field-based test therefore provides a much-needed alternative to allow for the assessment of aerobic capacity. Furthermore, validation of a field-based test could provide surfers, coaches, and sport scientists with a method of testing aerobic capacity, which could be used for preseason baseline testing, rehabilitation, and repeat fitness testing. As this study used a recreational cohort, further research needs to be conducted using a competitive surfing cohort.

### 4.2. Strength and Limitations

The key strengths of this study are the use of gold standard testing to determine VO_2peak_, whereby oxygen consumption was analyzed using an automated gas analysis system (Parvo Medics, TrueOne^®^, 2400, Sandy, UT, USA). Furthermore, to limit systematic bias between VO_2peak_ testing, randomization of ergometers was used. Finally, testing was conducted by three experienced exercise scientists under the supervision of a senior researcher with expertise in maximal aerobic and anaerobic testing of recreational and competitive surfers.

There are several limitations of this study which need to be recognized. Given the small sample size, it must be emphasized that these results cannot be generalized outside of this study. As the study included a small sample size and only recreational surfers, further research is needed using larger sample sizes and competitive surfers. Furthermore, standardization of board volumes (board volume based on anthropometrics) during the 400-m paddle test will allow for the validation of these preliminary results. Standardizing board volumes is imperative for future research given that board volumes have been shown to influence oxygen consumption [24].

## 5. Conclusions

This study demonstrated that two commercially available SWB ergometers can be effectively utilized when testing maximal aerobic capacity in a surfing cohort. The significant correlation of the two SWB ergometers and 400-m paddle test provide preliminary evidence to support the use of a 400-m paddle test as a suitable alternative when determining aerobic capacity. However, further research is needed with a larger sample and the standardization of board volumes. Collectively, these findings can assist surfers, coaches, and exercise scientists in identifying equipment beneficial in measuring maximal aerobic capacity and creating effective training programs, baseline testing, and rehabilitation.

## Figures and Tables

**Table 1:** Physical attributes and experience of recreational surfers M ± SD (n = 17).

Measure	Value (Mean ± SD)
Age (years)	34 ± 9
Height (cm)	176.79 ± 8.73
Mass (kg)	79.78 ± 11.32
Surfing Experience (years)	15.28 ± 8.67
Surfing Exposure (hours per week)	4.94 ± 2.36

**Table 2:** Key swim bench performance variables for recreational surfers M ± SD (n =17).

Measure	SwimFast SWB Ergometer	VASA SWB Ergometer	Mean Difference ± SD	t(df)	*p* Value
VO_2peak_ (L/min)	2.32 ± 0.60	2.30 ± 0.62	0.03 ± 0.22	0.46 (16)	0.63
VO_2peak_ (mL/kg/min)	29.24 ± 7.28	28.91± 7.16	0.33 ± 3.05	0.45 (16)	0.660
RER	1.24 ± 0.09	1.24 ± 0.09	−0.001 ± 0.06	−0.10 (14)	0.93
HR_peak_ (b·min^−1^)	170.5 ± 9.6	171.8 ± 9.9	−1.3 ± 8.7	−0.59 (14)	0.563
Peak aerobic power (W)	83.53 ± 28.05	89.41 ± 24.87	−5.88 ± 12.27	−1.98 (16)	0.066

Peak Volume of Oxygen Liters per minute—VO_2peak_ (L/min); Peak Volume of Oxygen milliliters per kilogram per minute; Volume of Carbon Dioxide—VCO_2_; Respiratory Exchange Ratio—RER; Heart Rate Peak beats per minute—HR_peak_ (b·min^−1^).
